# Radiation dose and fluoroscopy time of modern endovascular treatment techniques in patients with saccular unruptured intracranial aneurysms

**DOI:** 10.1007/s00330-020-06777-x

**Published:** 2020-03-19

**Authors:** Robert Forbrig, Yigit Ozpeynirci, Matthias Grasser, Franziska Dorn, Thomas Liebig, Christoph G. Trumm

**Affiliations:** 1 Institute of Neuroradiology, University Hospital, LMU Munich, Marchioninistrasse 15, 81377 Munich, Germany; 2 Siemens Healthineers, Forchheim, Germany

**Keywords:** Cerebral angiography, Endovascular procedures, Intracranial aneurysm, Radiation exposure

## Abstract

**Objectives:**

Modern endovascular treatment of unruptured intracranial aneurysms (UIAs) demands for observance of diagnostic reference levels (DRLs). The national DRL (250 Gy cm^2^) is only defined for coiling. We provide dosimetric data for the following procedures: coiling, flow diverter (FD), Woven EndoBridge (WEB), combined techniques.

**Methods:**

A retrospective single-centre study of saccular UIAs treated between 2015 and 2019. Regarding dosimetric analysis, the parameters dose area product (DAP) and fluoroscopy time were investigated for the following variables: endovascular technique, aneurysm location, DSA protocol, aneurysm size, and patient age.

**Results:**

Eighty-seven patients (59 females, mean age 54 years) were included. Total mean and median DAP (Gy cm^2^) were 119 ± 73 (89–149) and 94 (73; 130) for coiling, 128 ± 53 (106–151) and 134 (80; 176) for FD, 128 ± 56 (102–153) and 118 (90; 176) for WEB, and 165 ± 102 (110–219) and 131 (98; 209) for combined techniques (*p* > .05). Regarding the aneurysm location, neither DAP nor fluoroscopy time was significantly different (*p* > .05). The lowest and highest fluoroscopy times were recorded for WEB and combined techniques, respectively (median 26 and 94 min; *p* < .001). A low-dose protocol yielded a 43% reduction of DAP (*p* < .001). Significantly positive correlations were found between DAP and both aneurysm size (*r* = .320, *p* = .003) and patient age (*r* = .214, *p* = .046).

**Conclusions:**

This UIA study establishes novel local DRLs for modern endovascular techniques such as FD and WEB. A low-dose protocol yielded a significant reduction of radiation dose.

**Key Points:**

*• This paper establishes local diagnostic reference levels for modern endovascular treatment techniques of unruptured intracranial aneurysms, including flow diverter stenting and Woven EndoBridge device.*

*• Dose area product was not significantly different between endovascular techniques and aneurysm locations, but associated with aneurysm size and patient age.*

*• A low-dose protocol yielded a significant reduction of dose area product and is particularly useful when applying materials with a high radiopacity (e.g. platinum coils).*

## Introduction

Endovascular treatment of intracranial aneurysms has become a standard procedure since the International Subarachnoid Aneurysm Trial (ISAT) results confirmed at least equal clinical outcome when compared with neurosurgical approaches [1, 2]. As the guidelines for radiation protection have been updated recently [3], observance of diagnostic reference levels (DRLs) in endovascular treatment of intracranial aneurysms has increased in significance. The national DRL of the dose descriptor dose area product (DAP) defined by the Federal Office of Radiation Protection (250 Gy cm^2^) only refers to coil embolisation of intracranial aneurysms [4]; alternative modern endovascular treatment techniques such as extra-aneurysmal flow diverter (FD) stenting [5–7] or intraaneurysmal flow disruption (Woven EndoBridge (WEB) device) [8–10], that nowadays are routinely used, e.g. in broad-neck aneurysms, are not yet considered in the guidelines cited above.

As a consequence, published data on radiation dose often only take into account coil embolisation [11–15]. Furthermore, these studies mainly contain interventional data of unselected patients, i.e. patients with both elective and emergency aneurysm treatment (in case of a ruptured and/or symptomatic aneurysm).

Regarding endovascular treatment, a risk-benefit assessment is particularly essential in patients with an incidental, unruptured intracranial aneurysm (UIA). Recommendations on elective endovascular treatment of UIAs are (i) a high technical success and low peri-procedural complication rate, (ii) a reasonable radiation dose particularly in young patients according to the ALARA (as low as reasonably achievable) principle, and (iii) a limited intervention duration, as a prolonged fluoroscopy time is associated with an increased peri-procedural complication rate [16].

The aim of this retrospective single-centre study is the evaluation of radiation dose and fluoroscopy time in patients with an incidental saccular UIA, who underwent an elective aneurysm treatment using the following endovascular procedures: Coiling, FD, WEB, combined techniques. The provided data may be useful for the establishment of novel DRLs in the field of modern endovascular treatment of intracranial aneurysms.

## Material and methods

This retrospective single-centre study was approved by the responsible Institutional Review Board (project number 19-813) of the Ludwig-Maximilians-University Munich, Germany. The study was performed in accordance with the Declaration of Helsinki.

**Fig. 1:**
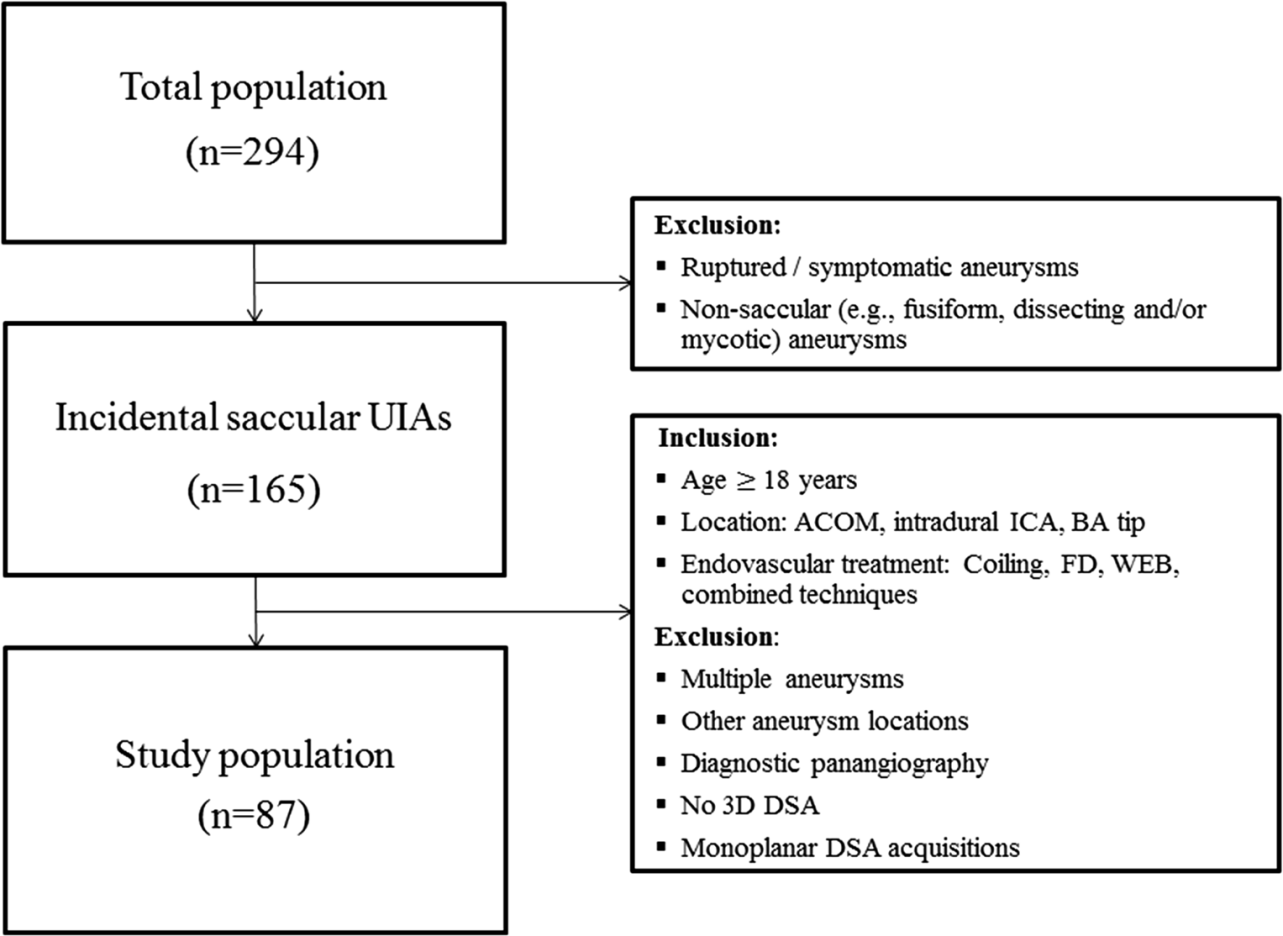
Flow chart of the inclusion and exclusion criteria. ACOM, anterior communicating artery; BA, basilar artery; DSA, digital subtraction angiography; FD, flow diverter; ICA, internal carotid artery; *n*, number; UIA, unruptured intracranial aneurysm; WEB, Woven EndoBridge device

We analysed patients with a saccular UIA who were endovascularly treated between January 2015 and May 2019. To increase the dosimetric homogeneity of the data pool and to reduce treatment bias, the following inclusion and exclusion criteria were defined (see flowchart in Fig. 1):

- Inclusion criteria:
  - Age ≥ 18 years
  - Location: anterior communicating artery (ACOM); intradural segments of the internal carotid artery (ICA), including posterior communicating artery and carotid T; tip of the basilar artery (BA)
  - Endovascular treatment: coiling, FD, WEB, combined techniques

- Exclusion criteria:
  - Ruptured and/or symptomatic aneurysms
  - Non-saccular (e.g. fusiform, dissecting, mycotic) aneurysms
  - Multiple aneurysms
  - Other intracranial aneurysm locations
  - Additional diagnostic pan- (four-vessel) angiography during the same intervention
  - No 3D angiography
  - Exclusively monoplanar digital subtraction angiography (DSA) acquisitions due to difficult working projection

Endovascular procedures were performed by five consultant neuroradiologists with six to more than 20 years of experience in interventional neuroradiology. The applied angiographic system was a biplane angiographic unit (Axiom Artis dBA, Siemens Healthineers). A transfemoral approach was used in each patient. Regarding the vessel visualisation, a non-ionic iodinated contrast agent was applied (iomeprol 300 mg iodine/ml; Imeron, Bracco Imaging). The angiographic workflow routinely comprised initial and final DSA acquisitions including arterial and venous phases on standard anteroposterior and lateral projections with a preferred field of view (FOV) of 32 cm, a 3D DSA with a FOV of 48 cm (or minimum of 42 cm) preset by the manufacturer, peri-procedural DSA acquisitions in arterial phase on working projections using a targeted FOV of 11 cm or 16 cm, and a pulsed fluoroscopy with a frame rate of 7.5 f/s. Regarding the DSA acquisition type, two protocols were preset by the manufacturer:

- Low dose (LD): 2 or 4 f/s (arterial phase), 1 f/s (venous phase), kV 73, pulse width 50 ms, dose 1820 μGy/p
- Normal dose (ND): 2 or 4 f/s (arterial phase), 1 f/s (venous phase), kV 73, pulse width 100 ms, dose 3000 μGy/p

### Radiation metrics

In each patient, all imaging data and dose reports retrieved from a dedicated picture archiving and communication system (syngo.via, Siemens Healthineers) were reviewed by two experienced neuroradiologists with 9 (R.F.) and 10 (C.G.T.) years of experience in diagnostic and interventional neuroradiology. In detail, the following parameters were documented: aneurysm size, aneurysm location, endovascular technique, DSA acquisition count, DSA protocol, fluoroscopy time and DAP (representing a surrogate measure of energy delivered to patients [15]), and DSA DAP. The individual total DAP was calculated by summing fluoroscopy and DSA DAP. Data of DSA acquisition count, fluoroscopy time, and DAP were documented by summing respective values of both X-ray tubes (biplane mode).

Furthermore, the impact of different DSA protocols on DAP was investigated. In detail, (1) the total DAP was compared between the LD, ND, and mixed-dose (MD; both LD and ND DSA acquisitions) groups, and (2) the individual dose index was calculated for each patient in the three groups, by using the following formula:

$$ \mathrm{Dose}\ \mathrm{index}=\mathrm{DSA}\ \mathrm{DAP}/\mathrm{DSA}\ \mathrm{acquisition}\ \mathrm{count} $$

### Statistics

Continuous data are provided as mean ± standard deviation (95% confidence interval) and/or median (25%; 75% interquartile range), and categorical data as counts and percent. Regarding the two outcome parameters DAP and fluoroscopy time, data were initially assessed for normality applying the Kolmogorov-Smirnov test considering the endovascular technique, aneurysm location, and DSA protocol. Variables were then compared according to the *t* test if data were normally distributed. The Mann-Whitney *U* test was used if data were not normally distributed. When statistically significant differences occurred, single posttest comparisons were performed by using the Mann-Whitney *U* test and *t* test with Bonferroni’s correction for multiple comparisons. To note, though DAP values were normally distributed, we also calculated and compared the respective median DAP for different endovascular techniques and aneurysm locations, enabling adjustment to the local DRL defined by the 75% percentile [17]. The Pearson correlation analysis was applied to investigate the impact of the two variables aneurysm size and patient age on radiation dose and fluoroscopy time, respectively. Data analysis was performed using IBM SPSS Statistics for Windows, Version 24.0 (IBM Corp.). A level of significance of *α* = 0.05 was used throughout the study.

## Results

### Patient characteristics

Patient characteristics are summarised in Table 1 and Fig. 1. We identified a total of 294 patients with either an UIA or ruptured/symptomatic intracranial aneurysm who have been treated endovascularly at our institution between January 2015 and May 2019. Eighty-seven out of 294 patients (29.6%; 59 females, mean age 54 years) had a saccular UIA and met further inclusion and exclusion criteria as described above. These 87 patients represent the study population.

**Table 1:** Characteristics of 87 patients with a saccular UIA undergoing endovascular treatment

Age, mean (range)	54 years (18–74)		
Sex	59 females (67.8%), 28 males (32.2%)		
Aneurysm size, median (range)	6.7 mm (2–30 mm)		
	ACOM (*n* = 30)	Intradural ICA (*n* = 40)	BA tip (*n* = 17)
Coiling (*n* = 26)	15/26 (57.7%)	8/26 (30.8%)	3/26 (11.5%)
FD (*n* = 24)	1/24 (4.2%)	23/24 (95.8%)	0/24 (0%)
WEB (*n* = 21)	11/21 (52.4%)	0/21 (0%)	10/21 (47.6%)
Combined (*n* = 16)	3/16 (18.75%)	9/16 (56.25%)	4/16 (25%)

*ACOM*, anterior communicating artery; *BA*, basilar artery; *FD*, flow diverter; *ICA*, internal carotid artery; *n*, number; *UIA*, unruptured intracranial aneurysm; *WEB*, Woven EndoBridge device

The median aneurysm size was 6.7 mm, with a minimum diameter of 2 mm (*n* = 3) and a maximum diameter of 30 mm (*n* = 1). To note, the three patients with the smallest UIA diameter were treated, as they had a history of subarachnoid haemorrhage due to rupture of another intracranial aneurysm, consequently harbouring a statistically increased risk of re-bleeding. Thirty out of 87 (34.5%) aneurysms were located at the ACOM, 40/87 (46%) at the intradural ICA, and 17/87 (19.5%) at the BA tip. Regarding the endovascular technique, 26/87 (29.9%) patients were treated by coiling (median 5 coils; range 1–21 coils), 24/87 (27.6%) by FD (1 device in 22/24 patients; 2 devices in 2/24 patients), 21/87 (24.1%) by WEB (1 device in 21/21 patients), and 16/87 (18.4%) by a combined technique (coiling + stent, *n* = 7; coiling + balloon remodeling, *n* = 2; coiling + FD, *n* = 5; WEB + stent, *n* = 1; FD + stent, *n* = 1). In detail, 15/30 (50%) ACOM aneurysms were treated by coiling, 1/30 (3.3%) by FD, 11/30 (36/7%) by WEB, and 3/30 (10%) by a combined technique (coiling + stent, *n* = 2; WEB + stent, *n* = 1). Intradural ICA aneurysms were treated by coiling in 8/40 (20%), using a FD in 23/40 (57.5%), and a combined technique in 9/40 patients (22.5%; coiling + stent, *n* = 2; coiling + balloon remodeling, *n* = 2; coiling + FD, *n* = 4; FD + stent, *n* = 1). BA tip aneurysms were treated by coiling in 3/17 (17.7%), using a WEB in 10/17 (58.8%), and a combined technique in 4/17 patients (23.5%; coiling + stent, *n* = 3; coiling + FD, *n* = 1).

### Radiation dose and fluoroscopy time

**Fig. 2:**
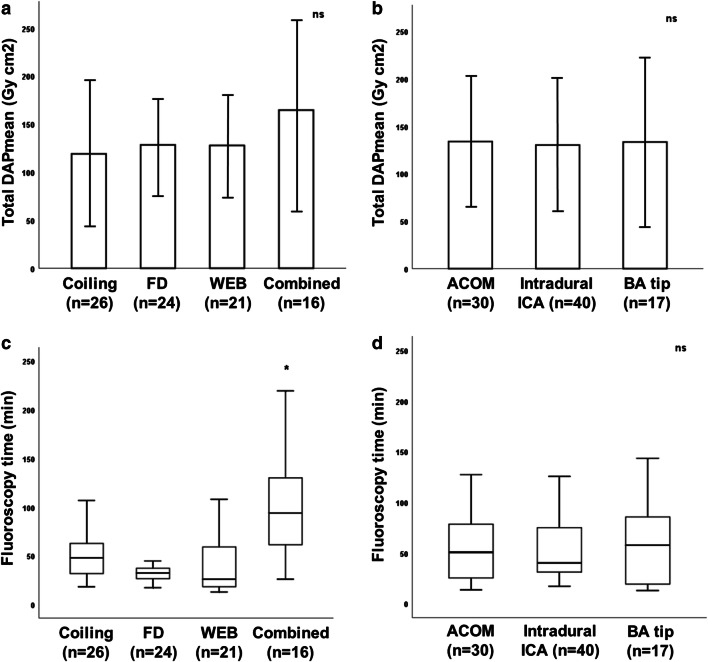
Total DAP and fluoroscopy time with regard to different endovascular techniques and aneurysm locations. Values are shown as mean ± standard deviation and median (25%; 75% percentile). Difference of mean total DAP did reach statistical significance when comparing neither the endovascular technique (**a**) nor the aneurysm location (**b**) (*p* > .05, each). Utilisation of a combined endovascular technique yielded a significant higher median fluoroscopy time in pairwise comparison to the three other groups (asterisk in **c**; *p* < .003, each), whereas median fluoroscopy time was not significantly different when comparing the aneurysm location (**d**; *p* > .05, each). DAP, dose area product; ACOM, anterior communication artery; BA, basilar artery; FD, flow diverter; ICA, internal carotid artery; min, minutes; *n*, number; ns, not significant; WEB, Woven EndoBridge device

**Fig. 3:**
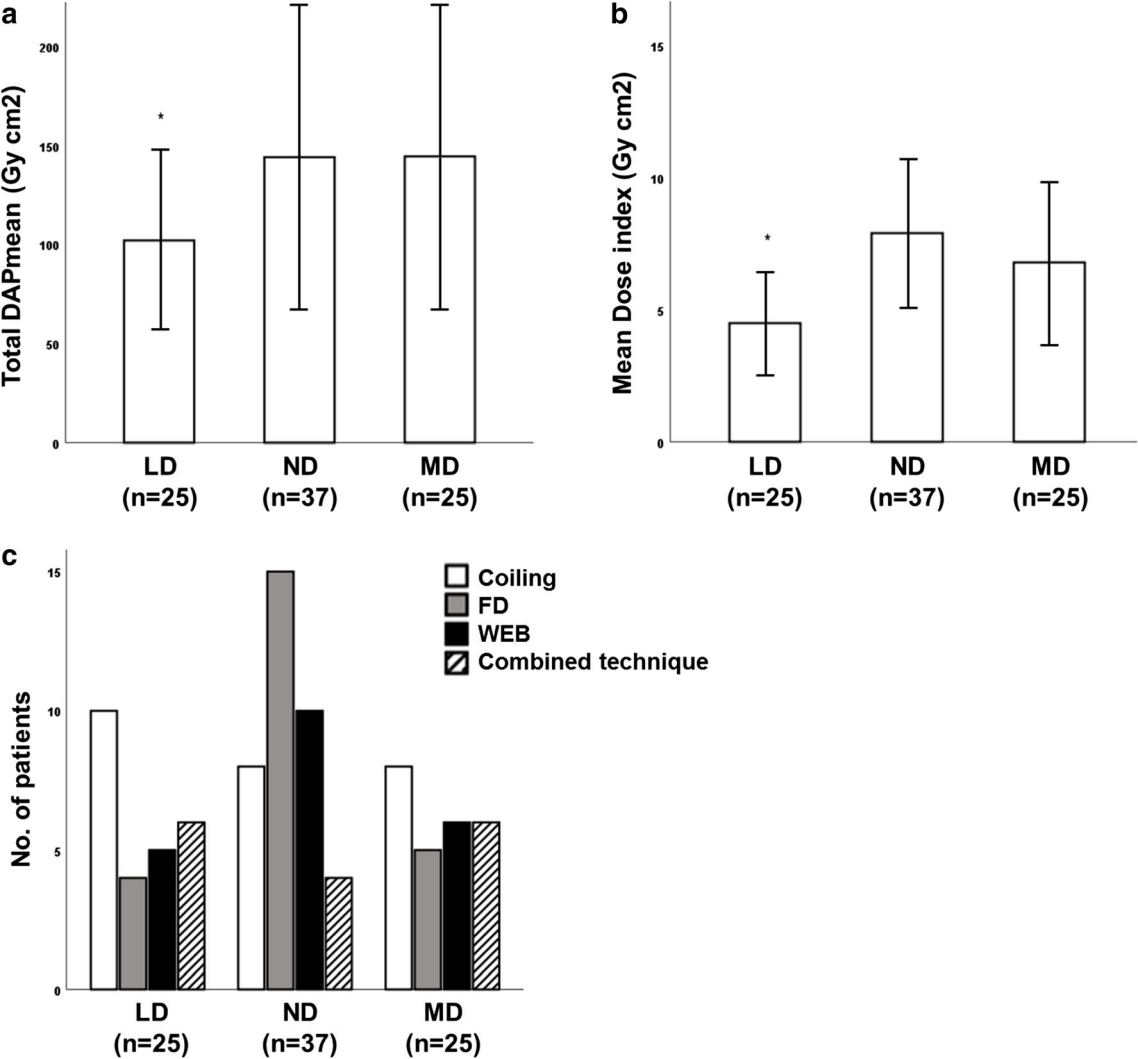
Total DAP and dose index with regard to different DSA protocols. Values are shown as mean ± standard deviation. Both mean total DAP and dose index were significantly lower in the LD group when compared with those in the ND and MD groups, respectively (asterisk in **a**, *p* < .023, each; and **b**, *p* < .001, each). A LD protocol was preferentially chosen in patients undergoing coil embolisation, whereas a ND protocol was most commonly applied in (**c**) FD and WEB cases. DAP, dose area product; DSA, digital subtraction angiography; LD, low dose; MD, mixed dose; *n*, number; ND, normal dose

Results of radiation dose and fluoroscopy time are illustrated in Table 2 and Figs. 2 and 3.

**Table 2:** DAP and fluoroscopy time regarding different endovascular techniques, aneurysm locations, and DSA protocols

Total DAP (*n* = 87) (Gy cm^2^)	130 ± 65 (116–144) (mean)		116 (78; 165) (median)		
Endovascular technique	Coiling (*n* = 26)	FD (*n* = 24)	WEB (*n* = 21)	Combined (*n* = 16)	*p* value
Mean DAP* (Gy cm^2^)	119 ± 73 (89–149)	128 ± 53 (106–151)	128 ± 56 (102–153)	165 ± 102 (110–219)	Coiling vs. FD: *p* = .550 Coiling vs. WEB: *p* = .591 Coiling vs. combined: *p* = .199 FD vs. WEB: *p* = .998 FD vs. combined: *p* = .277 WEB vs. Combined: *p* = .335
Median DAP^#^ (Gy cm^2^)	94 (73; 130)	134 (80; 176)	118 (90; 176)	131 (98; 209)	Coiling vs. FD: *p* = .085 Coiling vs. WEB: *p* = .203 Coiling vs. combined: *p* = .060 FD vs. WEB: *p* = .991 FD vs. combined: *p* = .547 WEB vs. combined: *p* = .542
FL time^#^ (min)	49 (32; 68)	34 (27; 44)	26 (18; 65)	94 (59; 133)	Coiling vs. FD: *p* = .267 Coiling vs. WEB: *p* = .061 Coiling vs. combined: *p = .002* FD vs. WEB: *p* = .087 FD vs. combined: *p = .001* WEB vs. combined: *p < .001*
Aneurysm location	ACOM (*n* = 30)	Intradural ICA (*n* = 40)	BA tip (*n* = 17)		
Mean DAP* (Gy cm^2^)	134 ± 68 (109–159)	130 ± 69 (108–152)	133 ± 87 (89–178)		ACOM vs. intradural ICA: *p* = .303 ACOM vs. BA tip: *p* = .173 Intradural ICA vs. BA tip: *p* = .505
Median DAP^#^ (Gy cm^2^)	116 (75; 178)	120 (80; 162)	110 (66; 172)		ACOM vs. intradural ICA: *p* = .410 ACOM vs. BA tip: *p* = .255 Intradural ICA vs. BA tip: *p* = .579
FL time^#^ (min)	51 (25; 80)	41 (31; 82)	58 (19; 90)		ACOM vs. intradural ICA: *p* = .491 ACOM vs. BA tip: *p* = .812 Intradural ICA vs. BA tip: *p* = .382
DSA protocol	LD (*n* = 25)	ND (*n* = 37)	MD (*n* = 25)		
Acquisition count* (*n*)	27 ± 13 (19–35)	28 ± 17 (19–37)	25 ± 13 (18–32)		LD vs. ND: *p* = .887 LD vs. MD: *p* = .637 ND vs. MD: *p* = .552
Mean DAP* (Gy cm^2^)	102 ± 45 (83–121)	144 ± 78 (118–170)	144 ± 77 (113–176)		LD vs. ND: *p = .018* LD vs. MD: *p = .022* ND vs. MD: *p* = .904
Mean dose index* (Gy cm^2^)	4.49 ± 1.76 (3.77–5.22)	7.89 ± 2.97 (6.90–8.88)	6.78 ± 3.06 (5.52–8.04)		LD vs. ND/MD: *p < .001*, *each* ND vs. MD: *p* = .159

Values of radiation dose, FL time, and acquisition count are summed for both X-ray tubes (biplane mode). Significant values with post hoc comparisons are indicated in italics

*ACOM*, anterior communicating artery; *BA*, basilar artery; *DAP*, dose area product; *DSA*, digital subtraction angiography; *FD*, flow diverter; *FL*, fluoroscopy; *ICA*, internal carotid artery; *LD*, low dose; *MD*, mixed dose; *min*, minutes; *n*, number; *ND*, normal dose; *ns*, not significant; *WEB*, Woven EndoBridge device

*Mean values were calculated using the *t* test and are shown as mean ± standard deviation (95% confidence interval)

^**#**^Median values were calculated using the Mann-Whitney *U* test and are shown as median (25%; 75% percentile)

Overall, the total mean and median DAP (Gy cm^2^) of 87 patients were 130 ± 65 (116–144) and 116 (78; 165), respectively. In detail, total mean and median DAP (Gy cm^2^) were 119 ± 73 (89–149) and 94 (73; 130) for coiling, 128 ± 53 (106–151) and 134 (80; 176) for FD, 128 ± 56 (102–153) and 118 (90; 176) for WEB, and 165 ± 102 (110–219) and 131 (98; 209) for combined techniques. We calculated the lowest mean and median DAP for the coiling group, and the highest mean DAP for the combined-technique group; however, pairwise comparison of total mean and median DAP between groups did not reach statistical significance (*p* > .05, each; Table 2). Concerning the aneurysm location, total mean and median DAP (Gy cm^2^) were 134 ± 68 (109–159) and 116 (75; 178) for ACOM aneurysms, 130 ± 69 (108–152) and 120 (80; 162) for intradural ICA aneurysms, and 133 ± 87 (89–178) and 110 (66; 172) for BA tip aneurysms (*p* > .05, each; Table 2).

Median fluoroscopy time was 49 min (32; 68) for coiling, 34 min (27; 44) for FD, 26 min (18; 65) for WEB, and 94 min (59; 133) for combined techniques. Median fluoroscopy time of the latter group was significantly higher when compared with the three other groups (*p* < .003, each; Table 2), with the biggest increment in comparison with the WEB group (*p* < .001). In contrast, pairwise comparison of median fluoroscopy time between the coiling, FD, and WEB groups was not significantly different (*p* > .05, each; Table 2). Regarding the aneurysm location, median fluoroscopy time was 51 min (25; 80) for ACOM aneurysms, 41 min (31; 82) for intradural ICA aneurysms, and 58 min (19; 90) for BA tip aneurysms (*p* > .05, each; Table 2).

A LD protocol was applied in 25/87 (28.7%), a ND protocol in 37/87 (42.5%), and a MD protocol in 25/87 patients (28.7%). Mean DSA acquisition count (biplane) did not significantly differ between groups (LD 27 ± 13 (19–35), ND 28 ± 17 (19–37), MD 25 ± 13 (18–32); *p* > .05, each; Table 2). Mean total DAP (Gy cm^2^) was 102 ± 45 (83–121) for the LD group, 144 ± 78 (118–170) for the ND group, and 144 ± 77 (113–176) for the MD group. The mean dose index (Gy cm^2^; mean DSA DAP/mean DSA acquisition count) was 4.49 ± 1.76 (3.77–5.22) for the LD, 7.89 ± 2.97 (6.90–8.88) for the ND, and 6.78 ± 3.06 (5.52–8.04) for the MD groups. Values were significantly lower in the LD group when compared with those in the ND and MD groups (mean total DAP: *p* = .018 and .022, respectively; mean dose index: *p* < .001, each), whereas difference of values between the ND and MD groups did not reach statistical significance (*p* = .159). According to the mean dose index, a LD protocol yielded a 43% reduction of DAP per DSA acquisition when compared with a ND protocol. A LD protocol was most commonly applied in patients undergoing coil embolisation (*n* = 10/25, 40%), whereas a ND protocol was preferentially chosen in FD (15/37, 40.5%) and WEB cases (10/37, 27%).

### Impact of aneurysm size and patient age on radiation dose and fluoroscopy time

Considering the entire study population (*n* = 87), we found a significantly positive correlation between the aneurysm size and both total DAP (*r* = .320, *p* = .003) and fluoroscopy time (*r* = .284, *p* = .008). Moreover, a significantly positive correlation was found between patient age and total DAP (*r* = .214, *p* = .046), whereas correlation between patient age and fluoroscopy time did not reach statistical significance (*r* = .122, *p* = .261).

## Discussion

In the present study, we provide detailed data of radiation dose and fluoroscopy time for modern endovascular treatment techniques in patients with saccular UIAs. With regard to the Euratom Basic Safety Standards (BSS) directive [18], we believe that our observed data may be substantial for the establishment of novel DRLs for modern techniques such as FD and WEB, as the existing national guidelines only provide DRLs for coiling (DAP 250 Gy cm^2^) [4]. Moreover, the indication for aneurysm treatment (elective or emergency) is not mentioned in these guidelines. In order to report dosimetric data of a standardised elective UIA treatment, data collection comprised the following angiographic algorithm: (1) catheterisation of the target vessel only, (2) initial biplane DSA run on standard anteroposterior and lateral projections, (3) 3D rotational angiography, (4) aneurysm treatment using the working projection and peri-procedural biplane control DSA runs, and (5) final biplane DSA run. We explicitly excluded patients with ruptured and/or symptomatic aneurysms, as an additional diagnostic cerebral four-vessel angiography during the same intervention is usually required in these cases (to detect/rule out further aneurysms), itself yielding a distinct amount of DAP [12–14, 19] and thus escalation of overall radiation dose. For example, Acton and colleagues [19] reported a median DAP of 74 Gy cm^2^ for a cerebral four-vessel angiogram. According to the Federal Office of Radiation Protection [4], we thus intended to report dosimetric data of the aneurysm treatment only.

According to the ICRP 135 publication [17], application of several radiation dose metrics (e.g. DAP and fluoroscopy time) is recommended for DRL establishment of fluoroscopically guided interventions. In this context, the DRL value is defined as the 75th percentile of the distribution of the DRL quantity [17], representing a commonly calculated radiation dose metric in neurointerventional procedures [11–15, 19–22]. In the present study, we observed a total mean and median DAP of 130 ± 65 (116–144) Gy cm^2^ and 116 (78; 165) Gy cm^2^, respectively. In detail, the calculated 75th percentile was 130 Gy cm^2^ for coiling, 176 Gy cm^2^ for each FD and WEB, and 209 Gy cm^2^ for combined techniques. The measured difference in radiation dose between the treatment groups clearly reflects the grade of aneurysm complexity, with a comparably lower DAP in simple coiling and a higher DAP in aneurysms treated by combined techniques; however, this difference did not reach statistical significance. To note, as the data pool was homogenised in order to reduce inter-individual dosimetric variations as described above, we indeed noted normally distributed DAP values, additionally enabling reliable report of the statistical mean considering the different endovascular techniques, aneurysm locations, and applied DSA protocols.

With regard to the literature, the median DAP for coiling was within the range of previously published data by other authors, e.g. Hassan et al 78.7 (59.5; 111.9) Gy cm^2^ [11] and Acton et al 100 (74; 123) Gy cm^2^ [19]. Furthermore, the slightly higher DAP values (when compared with coiling) in patients treated by FD, WEB, or a combined technique were still clearly below the values provided by recent dosimetric studies dealing with aneurysm embolisation [14, 20, 23, 24]. Moreover, as illustrated by other authors [13, 19, 20, 22], radiation dose metrics of fluoroscopically guided procedures are influenced by several confounders particularly in the field of interventional neuroradiology (e.g. complexity of procedures, tube settings and position, implementation of radiation reduction technologies, and experience of the medical staff); thus, DRLs should be defined locally for each centre.

Considering the aneurysm location, neither the DAP nor fluoroscopy time was significantly different when comparing the three most common anatomic sites treated in our institution (ACOM, intradural ICA, BA tip). We therefore assume that neither radiation dose nor fluoroscopy time is necessarily dependent on the aneurysm location as reported by Acton and colleagues [19], but rather on (1) the aneurysm size (as suggested by D’Ercole et al [13]) which itself more properly defines the choice of the dedicated endovascular technique and thus complexity of treatment, and (2) the anatomic approach which is often more sophisticated in elderly patients due to a commonly increased vessel tortuosity. In this context, we indeed found a significantly positive correlation between DAP and both aneurysm size (*r* = .320, *p* = .003) and patient age (*r* = .214, *p* = .046).

We observed a median fluoroscopy time of 49 min for coiling, 34 min for FD stenting, and 26 min for implantation of a WEB. These values are clearly in the range of published data on endovascular aneurysm embolisation [11–13]. In contrast, application of combined techniques in more complex aneurysms yielded a significantly higher fluoroscopy time (median 94 min) when compared with the solitary techniques, with the largest gap in comparison with WEB cases (*p* < .001).

With regard to the DSA acquisition mode, application of a LD protocol yielded a significantly lower DAP when compared with a ND protocol (mean DAP 102 versus 144 Gy cm^2^, *p* = .018). The impact of a LD protocol on radiation dose reduction was more objectively illustrated by calculating the dose index, which reflects the DAP per single DSA acquisition (mean dose index LD 4.49 Gy cm^2^ versus ND 7.89 Gy cm^2^, DAP reduction 43%; *p* < .001). A LD protocol was most commonly applied in aneurysms treated by coiling. Contrarily, a ND protocol was preferentially chosen in FD and WEB cases. This distribution in turn explains the slightly increased radiation dose in the FD and WEB groups when compared with the coiling group as illustrated above. However, the provided DRLs are still clearly below the official local DRL for coil embolisation [4]. Even though the choice of both the dedicated endovascular technique and DSA acquisition protocol is at the discretion of the interventional neuroradiologist, we believe that the following DSA protocol algorithm can be derived from our data, probably yielding—in addition to other techniques such as image noise reduction [22, 25]—further radiation dose optimisation in the field of endovascular UIA treatment:

1. The standard initial and final DSA runs of the relevant vascular territory (FOV 32 cm) should preferentially be conducted in LD mode, as this protocol is both appropriate for aneurysm visualisation and robust enough to detect/rule out catheter-associated complications such as thromboembolism, vasospasm, and arterial dissection.
2. Regarding peri-procedural targeted DSA runs in working projections (FOV 11–16 cm), application of a LD protocol is particularly useful in endovascular aneurysm treatment using materials with a high X-ray opacity (e.g. platinum coils). Contrarily, a ND protocol is reasonable when applying materials with a comparably lower fluoroscopic visibility (e.g. nitinol FD or WEB), allowing for a more detailed visualisation of the implanted device with respect to the aneurysmal sac and parent vessel.

As data reported in this study were retrospectively collected from only one neurovascular centre, our results have to be evaluated in light of several study limitations. First, neurointerventions were performed by usage of only one specific angiographic system from a single vendor (Siemens Healthineers). Second, the following parameters were not documented: peri-procedural change of strategy, size of the aneurysm neck, type of aortic arch. Third, several aneurysm locations were excluded due to procedural in-house management in our neurovascular centre (e.g. aneurysms of the middle cerebral artery are primarily treated by open neurosurgery) and/or rare occurrence (e.g. superior cerebellar artery, posterior cerebral artery); thus, dosimetric data observed in the present study cannot be generalised for all endovascular procedures. However, we believe that our selected study population may serve as a representative cohort of patients harbouring saccular UIAs at common anatomic sites accessible for endovascular treatment, as comparable data—particularly with regard to FD and WEB—are missing.

In conclusion, the present study introduces novel DRLs in the field of modern endovascular treatment of UIAs, including FD and WEB. Radiation dose was not significantly different between the endovascular procedures. However, radiation dose was comparably low in simple coiling and higher when using combined techniques, which are particularly applied in patients characterised by complex aneurysms. Aneurysm location did significantly alter neither radiation dose nor fluoroscopy time, whereas both aneurysm size and patient age were associated with radiation dose. Fluoroscopy time was the lowest for WEB and highest for combined techniques. A low-dose DSA protocol yielded a significant reduction of radiation dose and is particularly useful when applying high-opacity materials (e.g. platinum coils). With regard to the next Euratom version, we recommend a prospective collection of dosimetric data derived from multiple centres for definition of DRLs, considering different manufacturers and dose reduction techniques.

## Abbreviations

ACOM: Anterior communicating artery
BA: Basilar artery
BSS: Euratom Basic Safety Standards
DAP: Dose area product
DRL: Diagnostic reference level
DSA: Digital subtraction angiography
FD: Flow diverter
FOV: Field of view
ICA: Internal carotid artery
LD: Low dose
MD: Mixed dose
ND: Normal dose
SD: Standard deviation
UIA: Unruptured intracranial aneurysm
WEB: Woven EndoBridge device
