# Correction to: Radiation dose and fluoroscopy time of modern endovascular treatment techniques in patients with saccular unruptured intracranial aneurysms

**DOI:** 10.1007/s00330-021-07874-1

**Published:** 2021-05-03

**Authors:** Robert Forbrig, Yigit Ozpeynirci, Matthias Grasser, Franziska Dorn, Thomas Liebig, Christoph G. Trumm

**Affiliations:** 1 Institute of Neuroradiology, University Hospital, LMU Munich, Marchioninistrasse 15, 81377 Munich, Germany; 2 Siemens Healthineers, Forchheim, Germany

**Correction to: European Radiology (2020) 30:4504–4513**

10.1007/s00330-020-06777-x

The article “Radiation dose and fluoroscopy time of modern endovascular treatment techniques in patients with saccular unruptured intracranial aneurysms”, written by Robert Forbrig, Yigit Ozpeynirci, Matthias Grasser, Franziska Dorn, Thomas Liebig and Christoph G. Trumm, was originally published Online First without Open Access. After publication in volume 30, issue 8, page 4504–4513 the author decided to opt for Open Choice and to make the article an Open Access publication. Therefore, the copyright of the article has been changed to © The Author(s) 2020 and the article is forthwith distributed under the terms of the Creative Commons Attribution 4.0 International License, which permits use, sharing, adaptation, distribution and reproduction in any medium or format, as long as you give appropriate credit to the original author(s) and the source, provide a link to the Creative Commons licence, and indicate if changes were made. The images or other third party material in this article are included in the article’s Creative Commons licence, unless indicated otherwise in a credit line to the material. If material is not included in the article’s Creative Commons licence and your intended use is not permitted by statutory regulation or exceeds the permitted use, you will need to obtain permission directly from the copyright holder. To view a copy of this licence, visit http://creativecommons.org/licenses/by/4.0/.

